# Squamous cell carcinoma of the lung and pulmonary metastasis of papillary thyroid carcinoma: a case report

**DOI:** 10.1186/s13256-019-2177-6

**Published:** 2019-08-19

**Authors:** Arya Aminorroaya, Mohsen Khoshniatnikoo, Hossein Farrokhpour, Jamshid Vafaeimanesh, Mohammad Bagherzadeh

**Affiliations:** 1 Students’ Scientific Research Center, Tehran University of Medical Sciences, Tehran, Iran; 2 Universal Scientific Education and Research Network, Tehran, Iran; 3 Endocrinology & Metabolism Research Center, Endocrinology & Metabolism Clinical Sciences Institute, Tehran University of Medical Sciences, Dr. Shariati Hospital, Jalal Al Ahmad Highway, Tehran, 1411713137 Iran; 4 Gastroenterology & Hepatology Research Center, Qom University of Medical Sciences, Qom, Iran; 5 Clinical Research Development Center, Qom University of Medical Sciences, Qom, Iran

**Keywords:** Neoplasms, multiple primary, Thyroid cancer, papillary, Thyroid neoplasms, Carcinoma, squamous cell, Carcinoma, non-small cell lung, Lung neoplasms

## Abstract

**Background:**

The coexistence of malignancies in a patient may be explained by the tumor-to-tumor metastasis phenomenon or multiple primary malignant tumors, both of which are not common findings. Here, we are going to present a case with coexistent papillary thyroid carcinoma and primary squamous cell carcinoma of the lung.

**Case presentation:**

A 36-year-old Iranian man presented to our clinic for evaluation of constitutional symptoms. His past medical history was significant for papillary thyroid carcinoma due to which he had undergone total thyroidectomy, cervical lymph node dissection, and radioactive iodine therapy 14 years ago. Six months prior to admission, he received radioactive iodine therapy due to the metastatic involvement of both lungs with papillary thyroid carcinoma in another center with consequent improvement in symptoms. Diffuse nodular lesions in both lungs, a lesion in the lower lobe of his left lung, not present 6 months ago, peritoneal carcinomatosis, and several para-aortic lymphadenopathies were detected by imaging studies. A radioactive iodine uptake scan, positron emission tomography/computed tomography scan, and transbronchial biopsy of the lesion in the lung revealed concurrent squamous cell carcinoma of the lung and pulmonary metastasis of papillary thyroid carcinoma. After consultation with an oncologist, our patient received 6 months of chemotherapy; however, he died 8 months after presentation.

**Conclusions:**

Physicians should be aware of the possibility of the emergence of primary malignancies in patients with a history of papillary thyroid carcinoma, especially lung cancer as it is a common site of papillary thyroid carcinoma metastases. Using appropriate diagnostic evaluations in order to choose the best therapeutic option is of utmost importance.

## Background

Differentiated thyroid cancers arise from follicular epithelial cells and account for more than 90% of all thyroid neoplasms [1]. Among these, papillary thyroid carcinoma (PTC) is the most common type and contributed the most to the increase in the yearly incidence of thyroid cancer from 1975 to 2009, from 4.9 to 14.3 per 100,000, respectively [2]. Some investigators thought that this increase could be attributed to earlier detection of indolent tumors; however, others emphasized the role of under-recognized risk factors [3].

Lung cancer is the leading cause of cancer mortality and among the top three causes of disability-adjusted life-years, in both genders, across the world [4]. Non-small cell lung carcinoma, accounting for 85% of cases with this neoplasm, consists of squamous cell carcinoma (SCC), adenocarcinoma, and large cell carcinoma [5].

The coexistence of these malignancies in a patient may be explained by the tumor-to-tumor metastasis (TTM) phenomenon [6, 7] or multiple primary malignant tumors (MPMT) [8–10], both of which are not common findings. MPMT is characterized by suffering from two or more primary distinct neoplasms simultaneously, while TTM is defined as the coexistence of two or more primary tumors in the histopathologic specimen, and exclusion of imitative conditions including tumor emboli and direct invasion of adjacent neoplasm [11]. Here, we are going to present a case with coexistent PTC and primary SCC of the lung.

## Case presentation

A 36-year-old Iranian man presented to our clinic for evaluation of constitutional symptoms including involuntary weight loss, night sweating, and decreased appetite. He was known to have PTC for the past 14 years due to which he had undergone total thyroidectomy, cervical lymph node dissection, and radioactive iodine therapy (two times with a total dose of 325 mCi of ^131^I). Afterward, he abandoned his treatment and presented to another clinic with a chief complaint of a 3-month dry cough and bone pain, 6 months prior to admission. The evaluations were in favor of metastatic involvement of both lungs, ribs, and thoracic vertebrae with PTC. Consequently, he received 200 mCi of ^131^I and experienced improvement in symptoms. For suppression therapy of PTC, he received 0.1 mg of levothyroxine daily. Habitual history and familial history were unremarkable. In a lung examination, a fine crackle was auscultated in the lower lobe of his left lung. Moreover, there was an increased tactile fremitus in this region. In an abdominal examination, positive fluid wave and shifting dullness were detected indicating ascites. His physical examination was otherwise unremarkable.

He underwent an extensive workup. A chest computed tomography (CT) scan revealed diffuse nodular lesions in both lungs and a 3.5 × 4 cm lesion in the lower lobe of his left lung which was not present in the last imaging, 6 months prior to admission. A 47 × 32 mm hypoechoic lesion in his liver and free fluid in the peritoneal space were detected in abdominal ultrasonography. He then underwent a three-phasic abdominal CT scan that demonstrated that the aforementioned lesion was not consistent with hemangioma, while histological examination from CT-guided biopsy was diagnostic for hemangioma. The abdominal CT scan also showed a severe involvement of the omentum, peritoneal carcinomatosis, and several para-aortic lymphadenopathies. Ascites fluid had a low serum-ascites albumin gradient (< 1.1 g/dL); however, no malignant cells were detected in the cytological examination of two repeated specimens. Furthermore, several reactive lymph nodes, the biggest was 13.7 mm, were found in his left submandibular region in ultrasonography of his neck.

**Fig. 1:**
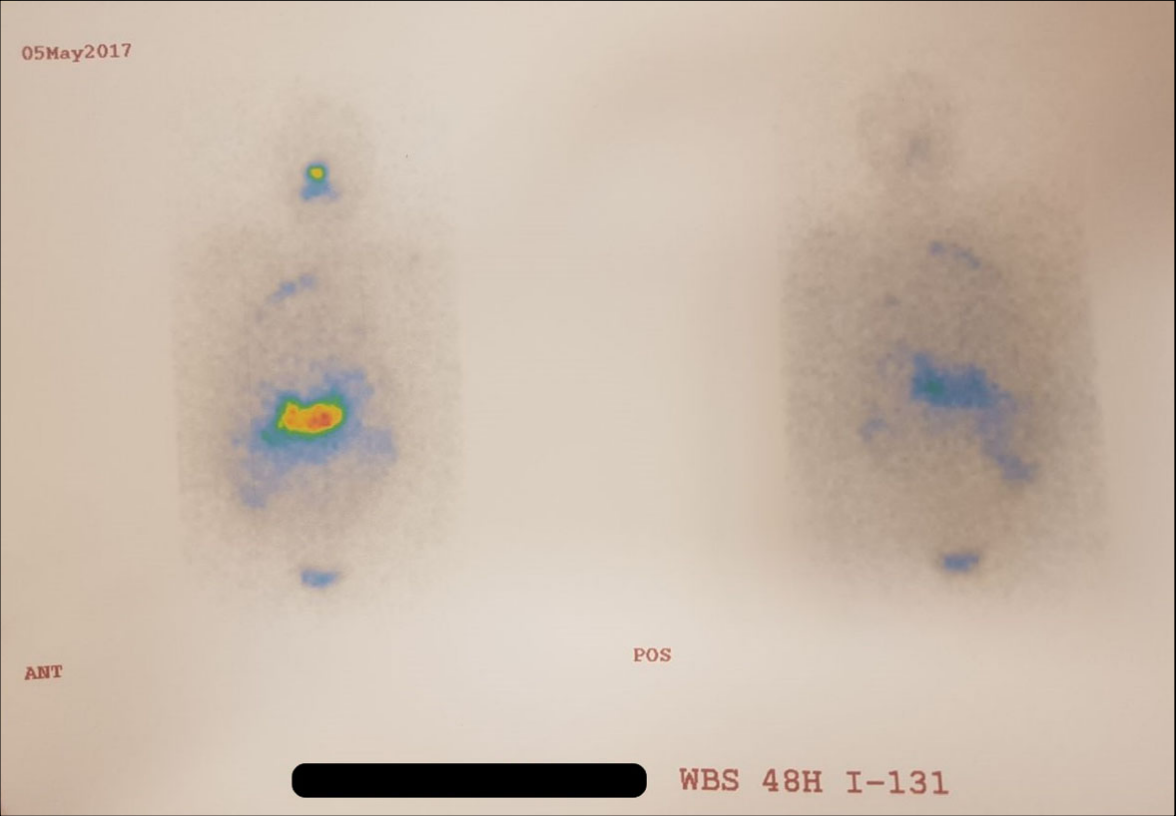
Imaging findings on radioactive iodine (^131^I) scan

**Fig. 2:**
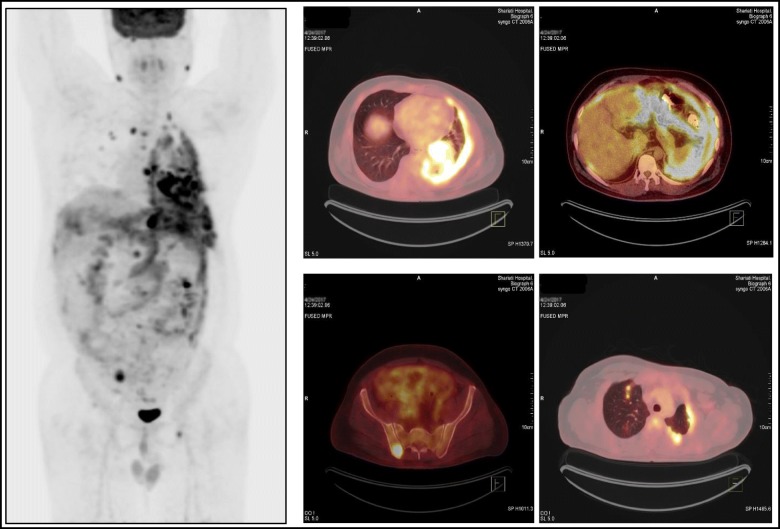
Imaging findings on fluorodeoxyglucose positron emission tomography/computed tomography scan

For evaluation of the lesions, a radioactive iodine uptake (RIU) scan was done which demonstrated several regions with high uptake in the upper lobe of his right lung and neck (Fig. 1). In the next step, a positron emission tomography/CT (PET/CT) scan revealed abnormal fluorodeoxyglucose (FDG)-avid regions in the lower lobe of his left lung (Fig. 2). Afterward, he underwent a flexible bronchoscopy for delineating the nature of the lesion through transbronchial biopsy. An immunohistochemical (IHC) examination of the specimen was focally positive for CK19, CK5/6, and P63 and negative for thyroglobulin and napsin, which is compatible with primary SCC of the lung.

All relevant laboratory data, including anti-thyroglobulin antibody, were in the normal range except for: thyroid-stimulating hormone, 100 mIU/L; erythrocyte sedimentation rate, 86 mm/hour; C-reactive protein, 3+; hemoglobin, 11 g/dL; and thyroglobulin, 70 ng/mL.

After consultation with an oncologist, our patient received 6 months of chemotherapy for SCC of the lung; however, he died 8 months later.

## Discussion and conclusions

In this case report, we present a coexistence of pulmonary metastasis of PTC and advanced SCC of the lung in a patient without any known risk factors of these malignancies.

In our patient, the positive RIU scan could be explained by PTC metastases; however, there are several reports regarding false-positive results due to other conditions including SCC of the lung [12, 13]. Moreover, FDG-avid regions in the PET/CT scan may be attributed to undifferentiated metastases of PTC [14] or primary lung neoplasm. Therefore, we could not ascertain the nature of the lesions from multi-modality imaging studies and tried to provide histopathological specimens by transbronchial biopsy. The IHC study was positive for CK5/6, P63, and CK19, in favor of SCC, and negative for napsin [15] and thyroglobulin which decreases the probability of TTM. Hence, mildly increased serum thyroglobulin (70 ng/mL) and the results of the RIU scan were attributed to undifferentiated pulmonary metastasis of PTC; however, we could not document it histopathologically because our patient refused to undergo further evaluations in the shared decision-making process.

Data from cancer registries show a prevalence of 0.4% to 21% for MPMT across the world [16, 17]. These studies demonstrated that the most common primary malignancy in this setting is head and neck cancer [16, 18]. Moreover, Babacan *et al.* reported head and neck cancer-lung cancer as the most common (13%) malignancy pair in men [18]. These findings may be explained by the higher survival of patients with head and neck cancer which provides them more time to develop other primary malignancies, in contrast with lung cancer [16].

There are scarce reports regarding the coexistence of PTC and primary lung cancer, both adenocarcinoma [8, 9] and SCC [10] of the lung. Acosta and Pins reported the case of a patient with concomitant PTC and SCC of the lung. Notably, they employed *BRAF* mutational analysis for differentiating MPMT from TTM [10]; however, we could not benefit from this method due to the lack of a pathological specimen of PTC metastasis.

In conclusion, physicians should be aware of the possibility of the emergence of primary malignancies in patients with a history of PTC, especially lung cancer as it is a common site of PTC metastases. In fact, the acceptable survival of patients with primary head and neck malignancies provides them more time to develop other primary neoplasms. Therefore, using appropriate diagnostic evaluations in order to make the correct diagnosis and choose the best therapeutic option is of utmost importance.

## Data Availability

All of the data and materials will be available upon request to the corresponding author.

## Abbreviations

CT: Computed tomography
FDG: Fluorodeoxyglucose
IHC: Immunohistochemical
MPMT: Multiple primary malignant tumors
PET: Positron emission tomography
PTC: Papillary thyroid carcinoma
RIU: Radioactive iodine uptake
SCC: Squamous cell carcinoma
TTM: Tumor-to-tumor metastasis
